# In vitro ruminal-microbial fermentation pattern: nutritional insights about some agricultural crop mesocarps (peel) in ruminant nutrition

**DOI:** 10.1186/s13568-023-01654-4

**Published:** 2023-12-19

**Authors:** Mohsen Kazemi

**Affiliations:** Department of Animal Science, Faculty of Agriculture and Animal Science, University of Torbat-e Jam, Torbat-e Jam, Iran

**Keywords:** Agricultural crop wastes, Melon species, Nutritional value, Chemical-mineral compositions

## Abstract

Different agricultural crop wastes (ACW) such as mesocarps (peel) are annually produced after consuming their edible parts in the world without any scientific information about their nutritional potential. Therefore, a study was conducted to determine the ruminal-microbial fermentation pattern and nutritional potential of some crop mesocarps (peel) including different species of melons (Watermelon, Galia melon, Rockmelon, Til sabz, and Til atashi) as well as cucumber and eggplant using common and standard laboratory methods. The amount of crude protein (CP) varied from 7.19 to 19.1% of dry matter (DM) for Til sabz and cucumber mesocarps, respectively. The highest (34.1% of DM) and lowest (15.4%) content of neutral detergent fiber (NDF) were related to eggplant and Til sabz peels, respectively (*p* < 0.05). The content of non-fiber carbohydrates (NFC) varied from 38.7% for eggplant peel to 66.6% for Til sabz. All mesocarps had a low DM percentage (4.88% of DM for cucumber to 8.45 for eggplant). Rockmelon peel had the highest amount of calcium (5.25 g/kg DM) and magnesium (5.75 g/kg DM) compared with the other mesocarps (*p* < 0.05). The highest amounts of potassium (33.4 g/kg of DM), sodium (7.15 g/kg of DM), and zinc (21 mg/kg of DM) were observed in the peels of watermelon, Til atashi, and cucumber, respectively (*p* < 0.05). The potential of ruminal-microbial gas production also differed from 55.6 ml/200 mg of DM for eggplant to 63.1 ml for Til sabz peel. Except for cucumber (8.75 MJ/kg of DM) and eggplant (8.71 MJ), other mesocarps (different melon species) had almost similar metabolizable energy (ME, 9.06–9.50 MJ/kg of DM). Among the studied mesocarps, the lowest ruminal-microbial DM and organic matter (OM) digestibility was also observed in eggplant and cucumber (*p* < 0.05). Gallia melon had the highest acid-base buffering capacity (267 mEq×10^− 3^, *p* < 0.05). According to our findings, the mesocarps of melons showed a higher nutritional value than the cucumber and eggplant. In general, Til sabz exhibited a notable favorite and superior nutritional characteristic compared with the other mesocarps.

## Introduction

A large population of small ruminants constitute an essential part of Iran’s rural economy, and more than 70% of their expenses are related to feed preparation. The increasing costs of common forages and grains are also impacting ruminant production. In fact, forage is the basis of the ruminant’s diet (such as corn silage, lucerne, or grass hay), and the concentrates provide additional protein and energy requirements (Rakita et al. 2021). All these parameters have driven animal nutritionists to formulate ruminant feed on cost-effective human-inedible ingredients that do not compete with human food. Using ACW in ruminant feeding is a common strategy in arid and semi-arid regions, especially Iran. Evaluation of the nutritional potential of ACW is also essential because they could make a vital contribution to animal nutrition. It has been reported that most of the cannery and fruit wastes could considered as excellent alternative feed resources in ruminant feeding (Bakshi and Wadhwa 2013). Bioactive compounds such as polyphenols and tannins are present in most ACW, which modulate ruminal microorganisms, and digestive-fermentative parameters, and help reduce greenhouse gas emissions (Branciari et al. 2021; Vastolo et al. 2022). The findings of Agbana et al. (2022) showed that watermelon mesocarp (rind) contains polyphenols that can compete favorably and even better than many common forages regarding nutritional parameters. In the study of Kazemi et al. (2019), it is reported that fresh or ensiled Iranian melon (*Cucumis melo* cv. Khatooni) wastes can be utilized in ruminant feeding as an alternative forage source; however, due to the low DM content and the risk of mildew in fresh wastes, ensiling with 2% grape vinegar could improve the nutritional potential and fermentative-digestive parameters. Also, in another study, a high nutritional potential for different parts (DM: 3.71–11.06%, CP: 16.3–24.7%, Ash: 8.43–20%, NDF: 16.8–23.7%, NFC: 32.9–53.1%) of a kind of melon plant has been reported recently (Kazemi et al. 2018). It was reported that eggplant forage as an unconventional feed can be used in diets of Yankasa rams during critical periods of feed scarcity without any adverse effects on growth performance and blood metabolites (Lakpini et al. 2015). It has been suggested that the feed blocks containing tomato and cucumber wastes can be successfully replaced by up to 35% of concentrate in the diet of dairy goats with reduced feed costs and methane production, increasing the proportion of polyunsaturated fatty acids in milk, and without compromising nutrient utilization or milk yield (Romero-Huelva et al. 2012). Oliveira et al. (2016) reported a discrete acidosis from volatile fatty acids in sheep receiving diets containing 25% melon up to 6 h post-consumption because of the sudden intra-ruminal delivery of high-Brix melon in non-adapted animals. The use of ACW as a cheap roughage resource has been the subject of serious research worldwide in recent decades. Hence, this study was designed to evaluate the nutritional potential and ruminal-microbial fermentation of mesocarps of some melon species as well as eggplant and cucumber in order to supply suitable nutritional data to improve the use of ACW in small ruminants feeding.

## Materials and methods

### Experiment location and sample collection

This experiment was conducted at the University of Torbat-e Jam (Latitude: 35° 14’ 38.4” N Longitude: 60° 37’ 21” E) in Razavi Khorasan Province, Iran. Different types of cucurbits fruits including watermelon, Galia Melon, rockmelon, Til sabz, and Til atashi, cucumber as well as a type of *Solanaceae* family including eggplant were purchased from different greengrocer’s shops in Torbet-e Jam, Iran. A keen knife was employed to separate several fruits’ mesocarps (peel) by hand. The prepared mesocarps were transferred immediately to the central laboratory for further analysis and DM determination. Til sabz and Til atashi are the local names of two Iranian melons (*Cucumis melo* L.).

### Laboratories measurements

The method of Jasaitis et al. (1987) was used for the determination of buffering capacity parameters and the sample’s pH. Briefly, 0.5 g of sample (DM basis) was weighted into a beaker, 50 mL of deionized distilled water was added, and then continuously stirred with a magnetic stir bar. Buffering capacity was determined by adding acid (0.1 N HCl) or base (0.1 N NaOH) until the pH increased to 4 or to 9, respectively. When the solution reached the equilibrium point after 3 min, the initial pH and all subsequent measurements were recorded. To determine the amount of DM, the fresh samples were transferred to an oven at 60 °C for 48 h until a constant weight was reached (AOAC 2005). The ether extract (EE) was determined according to the Soxhlet extract (AOAC 2005) method using the Soxhlet apparatus (Bakhshi, Iran). In determining the NFC values, the equation of Weiss et al. (1992) was used as follows: NFC (% of DM) = 100-(%NDF+%CP+%EE+%Ash). The ash content was determined by burning the samples in an electric furnace at 600 °C for 4 h. The NDF and acid detergent fiber (ADF) contents were determined using the reagents employed by Van Soest et al. (1991) and Ankom Technology (2006^a^, 2006^b^). The crude fiber (CF) was also determined using the Ankom system and method described by Komarek et al. (1996). The crude protein (CP) concentration was measured using the Kjeldahl (Bakhshi, V40, Tehran, Iran) method after acid digestion (Williams 1984). The concentration of minerals including sodium, calcium, magnesium, potassium, manganese, iron, and zinc was measured by atomic absorption spectrometry (SavantAA, GBC, Australia).

### The in vitro ruminal-microbial fermentation

The solutions for the in vitro medium were prepared according to the procedures described by Menke and Steingass (1988). The rumen fluid was collected 3 h after morning feeding from two fistulated sheep fed on a diet at the maintenance level. The taken rumen fluid was squeezed through four cheesecloth layers and kept in a water bath at 39 °C until the experiment started. The samples were ground through a 1 mm screen and about 200 mg of substrate plus strained rumen fluid and artificial saliva (ratio 1:2) were transferred into the 120 ml serum vials. The vials were sealed with rubber stoppers and aluminium caps, plumped with a crimper, and incubated in a water bath at 39 °C for 3, 6, 9, 12, 24, 48, 72, and 96 h. The method of Theodorou et al. (1994) was employed for running in vitro gas test and recording gas volume. The gas test was run in duplicate (five replications in each run). In each run, five serum vials without samples were considered as blank. A medium similar to that prepared for the microbial gas test was employed for the determination of total volatile fatty acids (TVFA), NH_3_–N, ruminal-microbial dry matter digestibility (DMD), ruminal-microbial organic matter digestibility (OMD), and pH following 24 h incubation. After 24 h incubation, each serum vial was opened, and its content was filtered via a Buchner funnel equipped with the polyester filter (45 μm pore size) and vacuum pump. The filtered residuals were poured into pre-weighed crucibles and oven-dried at 60 °C for 48 h. Finally, the DMD of each sample was calculated based on the amount of the initial sample weight (200 mg) and residual weight (Kazemi 2021). The fluid collected after filtration was used for TVFA, NH_3_–N, and pH determination. A standard and calibrated pH meter (Hana, Model HI 2210-01, USA) was employed for the determination of the pH of the culture medium after filtering. To determination of NH_3_–N, 5 ml of the filtered solution was mixed with 5 ml of 0.2 N HCl and stored in a freezer at − 18 °C for further analysis (Komolong et al. 2001). Sampling and preparation for the TVFA assay were performed according to the method provided by Getachew et al. (2004). The Markham device (Markham 1942) and protocol described by Barnett and Reid (1957) based on steam distillation were used for TVFA determination.

### Data analysis

The data were fitted to a nonlinear equation [$Y=b\left(1-{e}^{-ct}\right)]$of Ørskov and McDonald (1979) for the determination of in vitro gas production parameters. In that, *Y* = the gas production at time *t*, *b* = the potential of gas production (b_gas_, ml/200 mg DM), and *c* = the fractional rate of gas production (c_gas_, %/h). The data were analyzed in a completely randomized design using the GLM procedure of SAS (2002). All parameters were measured in five replicates. The equations described by Menke and Steingass (1988) were used for the determination of ME and net energy for lactation (NEl). Tukey’s test was used to determine the significance between each pair of means. The equation of Sanson and Kercher (1996) was employed for the determination of dry matter intake (DMI, % of body weight) as follow: $DMI=120/\%NDF$.

## Results

### The proximate analysis and minerals

Chemical compounds of some agricultural crop mesocarps (ACM) are presented in Table 1. Different ranges of chemical compounds were observed among the present mesocarps. The contents of CP ranged from 7.19% of DM in Til sabz to 19.1% in cucumber, DM from 4.88% of fresh weight in cucumber to 8.45 in eggplant, ADF from 9.63% of DM in Til sabz to 26.7% in eggplant, NDF from 15.4% of DM in Til sabz to 34.1 in eggplant, CF from 18.5% of DM in Til sabz to 33.7% in eggplant, EE from 1.97% of DM in Til sabz to 2.83 in eggplant, ash from 8.23% of DM in eggplant to 11.4% in cucumber, and NFC from 38.7% of DM in eggplant to 66.6% in Til sabz.

**Table 1:** Chemical compounds (% of DM) of some agricultural crop mesocarps

Item	DM	CP	ADF	NDF	CF	EE	Ash	NFC
Watermelon	5.58^e^	11.2^d^	16.3^d^	23.5^c^	25.2^bcd^	2.62^ab^	11.0^ab^	51.7^b^
Galia melon	7.25^c^	15.7^c^	15.8^d^	22.5^c^	23.5^cd^	2.08^bc^	10.2^c^	49.5^bc^
Rockmelon	8.14^ab^	9.45^e^	24.4^b^	30.0^b^	33.1^a^	2.36^abc^	10.6^bc^	47.6^c^
Til sabz	7.68^bc^	7.19^g^	9.63^e^	15.4^d^	18.5^d^	1.97^cd^	8.83^d^	66.6^a^
Til atashi	6.33^d^	7.58^f^	21.0^c^	28.0^b^	30.2^abc^	2.32^abc^	11.2^ab^	50.8^bc^
Cucumber	4.88^f^	19.1^a^	23.7^b^	29.2^b^	31.5^ab^	1.35^d^	11.4^a^	38.9^d^
Eggplant	8.45^a^	16.1^b^	26.7^a^	34.1^a^	33.7^a^	2.83^a^	8.23^d^	38.7^d^
SEM	0.24	0.96	1.24	1.29	1.27	0.11	0.26	1.95
*p*-value	< 0.0001	< 0.0001	< 0.0001	< 0.0001	< 0.0001	< 0.0001	< 0.0001	< 0.0001

Means within each column followed by a common letter are not significantly different at *p* < 0.05.

DM (% of fresh weight): dry matter; CP: crude protein; ADF: acid detergent fiber; NDF: neutral detergent fiber; CF: crude fiber; EE: ether extract; NFC: non-fiber carbohydrates.

Til sabz and Til atashi are the local names of two Iranian melons (*Cucumis melo* L.).

SEM: standard error of the mean.

The Mineral compositions of some ACM are exhibited in Table 2. Different contents of mineral compounds were observed among mesocarps. The concentrations of calcium varied from 1.31 g/kg of DM in watermelon to 5.25 g in rockmelon, potassium from 20.1 g/kg of DM in Til sabz to 33.4 g in watermelon, sodium from 1.93 g/kg of DM in eggplant to 7.15 g in Til atashi, magnesium from 1.67 g/kg of DM in watermelon to 5.75 g in rockmelon, iron from 89.6 mg/kg of DM in Til atashi to 127 mg in Galia melon, manganese from 7.25 mg/kg of DM in Til sabz to 17.3 mg in Galia melon, and zinc from 7.44 mg/kg of DM in Til sabz to 21 mg in cucumber.

**Table 2:** Mineral compositions of some agricultural crop mesocarps

Item	Ca	K	Na	Mg	Fe	Mn	Zn
Watermelon	1.31^d^	33.4^a^	3.53^c^	1.67^e^	78.7^c^	9.65^de^	14.4^bc^
Galia melon	3.65^b^	24.4^cd^	2.56^de^	3.69^bc^	127^a^	17.3^a^	13.2^c^
Rockmelon	5.25^a^	21.1^de^	2.60^cde^	5.75^a^	107^ab^	16.4^ab^	18.1^ab^
Til sabz	3.46^b^	20.1^e^	5.98^b^	3.35^bc^	99.8^bc^	7.25^e^	7.44^d^
Til atashi	3.41^b^	25.2^c^	7.15^a^	2.96^cd^	89.6^bc^	11.5^cd^	10.5^cd^
Cucumber	2.26^c^	31.5^ab^	2.96^cd^	3.99^b^	126^a^	13.8^bc^	21.0^a^
Eggplant	1.51^cd^	22.7^bc^	1.93^e^	2.28^de^	109^ab^	13.3^bc^	12.8^c^
SEM	0.29	1.07	0.41	0.28	3.90	0.77	0.98
*p*-value	< 0.0001	< 0.0001	< 0.0001	< 0.0001	< 0.0001	< 0.0001	< 0.0001

Means within each column followed by a common letter are not significantly different at *p* < 0.05.

Ca (g/kg DM): calcium; K (g/kg DM): potassium (g/kg DM); Na (g/kg DM): Sodium; Mg (g/kg DM): magnesium; Fe (mg/kg DM): Iron; Mn: manganese (mg/kg DM); Zn (mg/kg DM): Zinc.

Til sabz and Til atashi are the local names of two Iranian melons (*Cucumis melo* L.).

SEM: standard error of the mean.

### The in vitro ruminal-microbial fermentation

The gas test parameters obtained from the incubation of some ACM are shown in Table 3. There was a significant difference between the estimated parameters of the gas test. Til sabz produced more 96 h gas production (65.8 mL/200 mg of DM) than other mesocarps. The c_gas_ (0.110%/h) was also greatest in Til sabz (*p* < 0.05). The b_gas_ differed from 55.6 mL/200 mg of DM in eggplant to 63.1 mL in Til sabz.

**Table 3:** The gas test parameters obtained from the incubation of some agricultural crop mesocarps

Item	b_gas_	c_gas_	gas 12 h	gas 24 h	gas 48 h	gas 72 h	gas 96 h
Watermelon	57.7^b^	0.0838^b^	36.9^ab^	46.3^ab^	55.3^ab^	58.5^abc^	60.1^ab^
Galia melon	58.4^ab^	0.0826^b^	34.6^b^	45.4^abc^	54.4^b^	59.7^a^	62.0^ab^
Rockmelon	57.9^b^	0.0779^b^	35.3^ab^	45.4^abc^	54.8^ab^	58.2^abc^	60.8^ab^
Til sabz	63.1^a^	0.110^a^	35.8^ab^	49.5^a^	59.7^a^	62.8^a^	65.8^a^
Til atashi	58.1^b^	0.0742^b^	40.3^a^	49.1^a^	56.2^ab^	59.2^ab^	61.9^ab^
Cucumber	55.9^b^	0.0545^c^	28.2^c^	39.8^bc^	49.2^c^	54.5^bc^	58.1^b^
Eggplant	55.6^b^	0.0544^c^	27.9^c^	39.5^c^	48.9^c^	54.1^c^	57.7^b^
SEM	0.60	0.0041	1.02	0.94	0.86	0.71	0.68
*p*-value	0.003	< 0.0001	< 0.0001	0.0005	< 0.0001	0.0006	0.004

Means within each column followed by a common letter are not significantly different at *p* < 0.05.

b_gas_ (ml/200 mg DM): potential gas production; c_gas_ (%/h): fractional rate of gas production; gas 12, 24, 48, 72, and 96 h: the gas produced at times 12, 24, 48, and 72 h incubation.

Til sabz and Til atashi are the local names of two Iranian melons (*Cucumis melo* L.).

SEM: standard error of the mean.

The in vitro ruminal-microbial parameters obtained from the incubation of some ACM in a culture medium are presented in Table 4. A different range of fermentative-digestive parameters was observed among different mesocarps. Among the different peels, Til sabz had higher DMD (89.5%) and OMD (91.8%, *p* < 0.05). The amounts of DMD varied from 76.8% in eggplant to 89.5% in Til sabz, OMD from 79.6% in eggplant to 91.8% in Til sabz, NH_3_–N from 12.5 mg/dL in Til atashi to 13.6 mg in cucumber, TVFA from 74.4 mmol/L in eggplant to 79.6 in Til sabz, ME from 8.71 MJ/kg of DM in eggplant to 9.50 in Til sabz, NEl from 5.08 MJ/kg of DM in eggplant to 5.67 in Til sabz, pH from 6.37 in Til sabz to 6.52 in cucumber, and DMI from 3.52% of body weight in eggplant to 7.77 in Til sabz.

**Table 4:** The in vitro ruminal-microbial parameters obtained from the incubation of some agricultural crop mesocarps in a culture medium

Item	DMD	OMD	NH_3_–N	TVFA	ME	NEl	pH	DMI
Watermelon	87.4^a^	89.8^a^	12.8^bc^	78.0^ab^	9.31^ab^	5.52^ab^	6.48^ab^	5.11^b^
Galia melon	86.7^a^	89.0^a^	12.9^abc^	77.6^ab^	9.38^a^	5.57^a^	6.49^ab^	5.35^b^
Rockmelon	83.0ab	84.9^ab^	12.9^abc^	77.5^ab^	9.06^ab^	5.35^ab^	6.40^bc^	4.02^cd^
Til sabz	89.5^a^	91.8^a^	12.6^c^	79.6^a^	9.50^a^	5.67^a^	6.37^c^	7.77^a^
Til atashi	86.6^a^	89.1^a^	12.5^c^	79.3^a^	9.40^a^	5.60^a^	6.45^abc^	4.28^c^
Cucumber	77.8^b^	80.7^b^	13.6^ab^	74.6^b^	8.75^b^	5.12^b^	6.52^a^	4.11^cd^
Eggplant	76.8^b^	79.6^b^	13.5^a^	74.4^b^	8.71^b^	5.08^b^	6.43^abc^	3.52^d^
SEM	1.14	1.08	0.090	0.51	0.091	0.065	0.013	0.30
*p*-value	< 0.0001	< 0.0001	0.0005	0.0007	0.05	0.04	0.0024	< 0.0001

Means within each column followed by a common letter are not significantly different at *p* < 0.05.

DMD (%): dry matter digestibility following 24 h ruminal-microbial incubation; OMD (%): organic matter digestibility following 24 h ruminal-microbial incubation; NH_3_–N (mg/dL): ammonia nitrogen produced after 24 h ruminal-microbial incubation; TVFA (mmol/L): total volatile fatty acids produced after 24 h ruminal-microbial incubation; ME (MJ/kg DM): metabolizable energy; NEl (MJ/kg DM): net energy for lactation; pH: the amount of pH measured in the culture medium after 24 h incubation; DMI: dry matter intake (% of body weight).

Til sabz and Til atashi are the local names of two Iranian melons (*Cucumis melo* L.).

SEM: standard error of the mean.

### Buffering capacity

The pH and buffering capacity (mEq×10^− 3^) parameters of some ACM are presented in Table 5. The highest content of acid-base buffering capacity (267 mEq×10^− 3^) and base-buffering capacity (104 mEq×10^− 3^) were observed in Galia Melon (*p* < 0.05). Cucumber exhibited the highest contents of titratable acidity (365 mEq×10^− 3^), acid-buffering capacity (171 mEq×10^− 3^), and plant’s pH (6, *p* < 0.05). The titratable alkalinity (359 mEq×10^− 3^) was greatest in eggplant (*p* < 0.05).

**Table 5:** The pH and buffering capacity (mEq×10^− 3^) parameters of some agricultural crop mesocarps

Item	Acid-base buffering capacity	Titratable alkalinity	Base-buffering capacity	Titratable acidity	Acid-buffering capacity	pH
Watermelon	171^e^	212^d^	60.0^d^	251^b^	111^d^	5.46^c^
Galia melon	267^a^	342^b^	104^a^	262^b^	163^a^	5.70^b^
Rockmelon	167^e^	217^d^	60.8^d^	221^c^	106^d^	5.43^c^
Til sabz	197^d^	195^e^	48.3^e^	143^e^	148^b^	4.97^e^
Til atashi	141^f^	195^e^	50.6^e^	167^d^	90.0^e^	5.14^d^
Cucumber	248^b^	231^c^	76.9^c^	365^a^	171^a^	6.00^a^
Eggplant	221^c^	359^a^	85.6^b^	94f	136^c^	4.80^f^
SEM	9.52	14.47	4.19	18.6	6.36	0.087
*p*-value	< 0.0001	< 0.0001	< 0.0001	< 0.0001	< 0.0001	< 0.0001

Means within each column followed by a common letter are not significantly different at *p* < 0.05.

Til sabz and Til atashi are the local names of two Iranian melons (*Cucumis melo* L.).

SEM: standard error of the mean.

## Discussion

The use of ACW as an alternative animal feed has been considered for the last decades. There is a great variation in their chemical composition, which is due to several factors such as plant or geographical origin, treatments during harvesting and processing, or climatic conditions during their cultivation. In the present study, a considerable variation among the mesocarps used in this study was observed in chemical composition. The difference in chemical composition among mesocarps was in line with previous studies (Mirzaei-Aghsaghali & Maheri-Sis 2008; Abbeddou et al. 2011). The CP concentration of melon’s mesocarps was lower than those reported for alfalfa (Kazemi and Valizadeh 2019). The lower content of DM in melon peel species (5.58–8.14% of fresh weight vs. 9.2%) is in agreement with the finding of Zhivkova (2021). The amounts of CP, ash, and CF for watermelon peel have been reported to be about 12.4 (vs. 11.2%), 5.03 (11%), and 26.3% of DM (vs. 25.2%), respectively (Dibanda Romelle et al. 2016). In line with our finding, a high content of CP (26.5% DM vs. 19.1%) has been reported for cucumber mesocarp (peel) by Henry Niyi et al. (2019). The CP content of some melons including rockmelon (9.45% DM), Til sabz (7.19%), and Til atashi (7.58%) is relatively low similar to those reported for corn silage (7.93–8.12% DM) by De Melo et al. (2023). The content of EE (1.35–2.83% of DM) for mesocarps here was comparable with that reported for *Medicago sativa* (1.76% of DM) by Kazemi and Valizadeh (2019).

Minerals are needed for normalizing the body’s metabolic processes in small ruminants. The content of iron in mesocarps was above the levels of 50 mg/kg proposed as adequate for grazing animals (Mirzaei 2012). Also, in the present study, the level of potassium in all mesocarps (20.1–33.4 g/kg DM) was above 8 g/kg DM recommended for grazing animals (Mirzaei 2012). It has, however, been reported that some high-producing ruminants under heat stress may require potassium level above 10 g/kg. The range of manganese in mesocarps (7.25–17.3 mg/kg DM) was below the critical level of 40 mg/kg for meeting the manganese requirements of ruminants (Mirzaei 2012). An adequate range of 1–4 g sodium/kg DM (vs. 1.93–7.15 g/kg DM in the mesocarps) has been recommended for ruminants (Underwood 1981). It has been reported that 30 mg/kg is a critical level of dietary zinc, although it has been recommended that concentrations of 12–20 mg zinc/kg DM are sufficient for fattening ruminants (ARC 1980; Mirzaei 2012). The calcium requirement for maintenance, growth, and lactation in sheep has been reported to be about 1200–2600 mg/kg DM (Reuter and Robinson 1997). In this regard, the calcium content of mesocarps was in the range of 1.31 g/kg DM for watermelon to 5.25 g/kg DM for rockmelon. The range of minerals needed in sheep feeding is about 0.50–0.80 g/kg DM potassium, 0.09–0.18 sodium, 0.20–0.82 calcium, 0.16–0.38 phosphorus, and 0.12–0.18 magnesium (Moniello et al. 2005). Therefore, the present mesocarps are easily able to supply the mineral requirements of sheep.

The in vitro gas test technique, either based on volume or pressure measurements, was primarily set up for the estimation of the rate and extent of fermentation of concentrates or forages in ruminants (Amanzougarene and Fondevila 2020). Using in vitro gas test method has become a widespread alternative to overcome the labor, cost, and time costs of in vivo tests (Kazemi and Valizadeh 2019; Kazemi 2019; Kazemi et al. 2019; Azizi et al. 2020; Kazemi 2020; Kazemi and Valizaeh 2020; Kazemi 2021). At the same time, they fit animal welfare considerations better than in vivo experiments (Amanzougarene and Fondevila 2020). Gas production reflects the generation of short-chain fatty acids and microbial mass in a culture medium (Getachew et al. 1998). Incubation of Til sabz compared with other mesocarps resulted in greater b_gas,_ c_gas_, and 24, 48, 72 h gas production. This can be explained by the fact that the ruminal microorganisms can better digest Til sabz due to its lower NDF and ADF compared with other mesocarps. A negative relation between b_gas_ and the fiber content has been reported by Kazemi (2019).

In the present study, an increase in DMD, OMD, and TVFA of Til sabz compared with other mesocarps can be attributed to the lower content of fibers (NDF, ADF, and CF), whereas a strong positive relation between TVFA, DMD, and OMD has been also reported by Kazemi and Valizadeh (2019). On the other hand, this shows Til sabz due to its lower NDF and ADF contents and higher DMD and OMD exhibits higher TVFA in the culture medium compared with other mesocarps. It has been reported that DMD provide a poor indication of true fermentability (McSweeney et al. 1999). Instead, the authors recommended measuring end products of in vitro fermentation such as ammonia, short and branched chain fatty acids to better assess the nutritional value of forage plants (Pérez-Márquez et al. 2023). In this regard, the present study shows a suitable concentration of TVFA in different melons’ mesocarps. The OMD and TVFA in Til sabz were higher than those other mesocarps, possibly because of its higher NFC contents, which is a vital substrate for the growth of ruminal microorganisms (Anele et al. 2009). Estimating ME value ​​is essential in ration formulation purposes and determining the economic value of feedstuffs for other goals (Getachew et al. 2000). The ME (8.71–9.50 MJ/kg DM) and NEl (5.08–5.67 MJ/kg DM) of different mesocarps were comparable with that reported for *Medicago sativa* (ME: 9.01 MJ/kg DM, NEl: 5.36 MJ/kg DM) by Kazemi and Valizadeh (2019).

Determination of the buffering capacity of feedstuffs for ruminants had a functional role in a ruminal pH balance. Introducing crop wastes with a favorite buffering capacity might enhance nutrient digestibility in the digestive tract, improve the digestive tract’s health and increase the ruminant performance. In the present work, it was observed different buffering capacities among different mesocarps which can be attributed to their different chemical compositions. It has been reported that the initial pH and titratable acidity of feedstuff ingredients are the most critical determinants of ruminal pH (Kazemi et al. 2023). Based on the present finding, the highest titratable acidity was observed in the cucumber peel, indicating high resistance to acidification. Also, a higher acid-base buffering capacity in Galia melon indicates the high capacity for balancing the ruminal pH. In conclusion, as a result of ruminal-microbial fermentation and proximate analysis, it can be concluded that melon groups had a relatively higher nutritional value compared with the cucumber and eggplant mesocarps. Furthermore, Til sabz exhibited the highest nutritional value among different mesocarps. The nutritional value of present mesocarps can be comparable with alfalfa. In general, the present mesocarps can be accounted as a suitable substitution for common forages in small ruminants feeding during feed scarcity. In the future, further research is recommended to evaluate the nutritional potential in vivo.

## Data Availability

The data will be made available upon request.
